# Supplementary material for: Bayesian refinement of protein structures and ensembles against SAXS data using molecular dynamics
Source: PLoS Comput Biol. 2017 Oct 18;13(10):e1005800. doi: 10.1371/journal.pcbi.1005800 (PMC5662244; doi:10.1371/journal.pcbi.1005800)
Supplement: S1 Archive — (BZ2) [file pcbi.1005800.s011.tar.bz2 › gromacs_4.62_bayesian_saxs_refi/share/html/online/g_h2order.html]

g\_h2order


| g\_h2order Main Table of Contents |

**VERSION 4.6  
Sat 19 Jan 2013**

---

### Description

g\_h2order computes the orientation of water molecules with respect to the normal
of the box. The program determines the average cosine of the angle
between the dipole moment of water and an axis of the box. The box is
divided in slices and the average orientation per slice is printed.
Each water molecule is assigned to a slice, per time frame, based on the
position of the oxygen. When -nm is used, the angle between the water
dipole and the axis from the center of mass to the oxygen is calculated
instead of the angle between the dipole and a box axis.

### Files

| option | filename | type | description |
| --- | --- | --- | --- |
| **-f** | traj.xtc | Input | Trajectory: xtc trr trj gro g96 pdb cpt |
| **-n** | index.ndx | Input | Index file |
| **-nm** | index.ndx | Input, Opt. | Index file |
| **-s** | topol.tpr | Input | Run input file: tpr tpb tpa |
| **-o** | order.xvg | Output | xvgr/xmgr file |

### Other options

| option | type | default | description |
| --- | --- | --- | --- |
| **-[no]h** | bool | no | Print help info and quit || **-[no]version** | bool | no | Print version info and quit || **-nice** | int | 19 | Set the nicelevel || **-b** | time | 0 | First frame (ps) to read from trajectory || **-e** | time | 0 | Last frame (ps) to read from trajectory || **-dt** | time | 0 | Only use frame when t MOD dt = first time (ps) || **-[no]w** | bool | no | View output .xvg, .xpm, .eps and .pdb files || **-xvg** | enum | xmgrace | xvg plot formatting: xmgrace, xmgr or none || **-d** | string | Z | Take the normal on the membrane in direction X, Y or Z. || **-sl** | int | 0 | Calculate order parameter as function of boxlength, dividing the box in this number of slices. |

### Known problems

- The program assigns whole water molecules to a slice, based on the first atom of three in the index file group. It assumes an order O,H,H. Name is not important, but the order is. If this demand is not met, assigning molecules to slices is different.

---

http://www.gromacs.org  
gromacs@gromacs.org
